# Predicting COVID-19 Incidence Through Analysis of Google Trends Data in Iran: Data Mining and Deep Learning Pilot Study

**DOI:** 10.2196/18828

**Published:** 2020-04-14

**Authors:** Seyed Mohammad Ayyoubzadeh, Seyed Mehdi Ayyoubzadeh, Hoda Zahedi, Mahnaz Ahmadi, Sharareh R Niakan Kalhori

**Affiliations:** 1 Department of Health Information Management School of Allied Medical Sciences Tehran University of Medical Sciences Tehran Iran; 2 Department of Electrical and Computer Engineering McMaster University Hamilton, ON Canada; 3 School of Health Management and Information Sciences Iran University of Medical Sciences Tehran Iran; 4 Department of Pharmaceutics School of Pharmacy Shahid Beheshti University of Medical Sciences Tehran Iran

**Keywords:** coronavirus, COVID-19, prediction, incidence, Google Trends, linear regression, LSTM, pandemic, outbreak, public health

## Abstract

**Background:**

The recent global outbreak of coronavirus disease (COVID-19) is affecting many countries worldwide. Iran is one of the top 10 most affected countries. Search engines provide useful data from populations, and these data might be useful to analyze epidemics. Utilizing data mining methods on electronic resources’ data might provide a better insight into the COVID-19 outbreak to manage the health crisis in each country and worldwide.

**Objective:**

This study aimed to predict the incidence of COVID-19 in Iran.

**Methods:**

Data were obtained from the Google Trends website. Linear regression and long short-term memory (LSTM) models were used to estimate the number of positive COVID-19 cases. All models were evaluated using 10-fold cross-validation, and root mean square error (RMSE) was used as the performance metric.

**Results:**

The linear regression model predicted the incidence with an RMSE of 7.562 (SD 6.492). The most effective factors besides previous day incidence included the search frequency of handwashing, hand sanitizer, and antiseptic topics. The RMSE of the LSTM model was 27.187 (SD 20.705).

**Conclusions:**

Data mining algorithms can be employed to predict trends of outbreaks. This prediction might support policymakers and health care managers to plan and allocate health care resources accordingly.

## Introduction

Recently, a respiratory disease originating from coronavirus occurred in Wuhan City of China. Since the first positive case of this virus was in 2019, this coronavirus was named coronavirus disease (COVID-19) by the World Health Organization (WHO) [1]. Some hypotheses attribute the origin of this virus to seafood and bats [2].

COVID-19 has spread globally and has affected most countries; it was defined as a pandemic by the WHO in March 2020 [3]. As of March 21, 2020, COVID-19 has affected 186 countries and territories around the world, with more than 280,000 confirmed cases and 11,842 deaths [4]. Iran is one of the top 10 countries affected by this virus [4].

As COVID-19 is spreading rapidly worldwide, prediction models can help in health resource management and planning for prevention purposes. Google search data is one information resource that contains useful information to predict and estimate epidemics [5]. Data mining algorithms and techniques are well-known tools for predictive model development and data analysis. They can implicitly extract useful information from raw data [6-8]. The extracted knowledge can be used in different areas such as the health care industry. Recently, a large amount of data was generated in health care, including those on patients, diseases, and diagnoses.

The tasks in data mining fall into two categories: (1) descriptive tasks that deal with the general properties of the data and (2) predictive tasks, wherein the goal is to build models that can estimate the mapping from inputs to outputs by using a sample of data called training data. The trained models can be deployed to make predictions of the outputs for unseen inputs. These techniques are more flexible and efficient for exploratory analysis than the traditional statistical analysis [9].

In this study, data mining models were used to build predictive models from Google search data to predict the incidence of COVID-19 in Iran.

## Methods

### Dataset

The daily new cases of coronavirus (daily incidence) from February 15, 2020, to March 18, 2020, in Iran were obtained from the Worldometer website [10].

Google Trends [11] was searched for concepts related to COVID-19, from February 10, 2020, to March 18, 2020. The related concepts were suggested by one of the authors. A dataset consisting of 10 input features including the previous day’s search trends, cases of the previous day, and a target value (new cases of that day) was created. The total number of entries was calculated for the 37 days. The list of features is shown in Table 1. The terms in square brackets were searched in the corresponding Persian language words. The “pd” postfix in the features’ name indicates that the features are related to the previous day.

Google Trends does not provide absolute search numbers but instead, provides a measure entitled interest over time, which is described as “A value of 100 is the peak popularity for the term. A value of 50 means that the term is half as popular. A score of 0 means there was not enough data for this term” [11]; for consistency, the values of the daily new cases were transformed to the range between 0 to 100.

**Table 1:** Features used for predicting new COVID-19 cases.

Feature name	Description
[Corona]_pd	The interest of “Corona” search term in Persian for the previous day in Iran
COVID-19_pd^a^	The interest of “COVID-19” search term for the previous day in Iran
Coronavirus_pd	The interest of “Coronavirus” topic for the previous day in Iran
[Antiseptic selling]_pd	The interest of “Antiseptic selling” search term in Persian for the previous day in Iran
[Antiseptic buying]_pd	The interest of “Antiseptic buying” search term in Persian for the previous day in Iran
[Hand washing]_pd	The interest of “Handwashing” search term in Persian for the previous day in Iran
Hand sanitizer_pd	The interest of “Hand sanitizer” topic for the previous day in Iran
Ethanol_pd	The interest of “Ethanol” topic for the previous day in Iran
Antiseptic_pd	The interest of “Antiseptic” topic for the previous day in Iran
Cases_pd	COVID-19 Incidence of the previous day in Iran
New cases	COVID-19 Incidence of prediction day in Iran (Label)

^a^COVID-19: coronavirus disease

### Modeling and Evaluation

#### Linear Regression

One of the data mining techniques used for prediction tasks is linear regression. In a problem with one predictor, this technique tries to find the best line to fit. That line could relate the predictor and prediction values. The extended version of this one-predictor regression is called multiple linear regression and is used for multiple-predictor problems [12]. We used this type of linear regression in this study.

#### Long Short-Term Memory

Long short-term memory (LSTM) is an artificial recurrent neural network that is an effective model for the prediction of time series where data are sequential [9]. By storing the past in hidden states, they can predict the outputs more accurately. In this study, the aim was to estimate the number of positive COVID-19 cases through time; as this is a well-suited task for the LSTM model, we used this model in our study.

**Figure 1:**
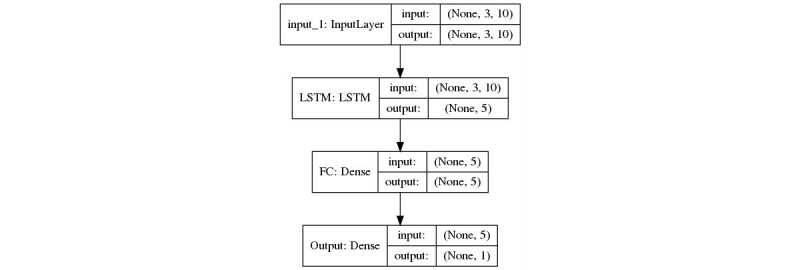
Proposed LSTM network architecture. LSTM: long short-term memory.

The linear regression model and a 3-layer LSTM model (Figure 1) are employed to predict the daily new cases. RapidMiner Studio 9.3.001 (RapidMiner GmbH) and Python 3.7.3 (Python Software Foundation) were used for modeling and evaluation. Tensorflow (Google Brain Team) and Keras (François Cholle) were used as frameworks for training LSTM models. In addition, 10-fold cross-validation was used to evaluate the performance of the models, and the root mean square error (RMSE) metric was chosen for performance evaluation:

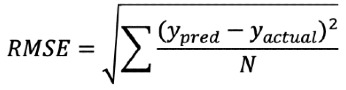



## Results

**Figure 2:**
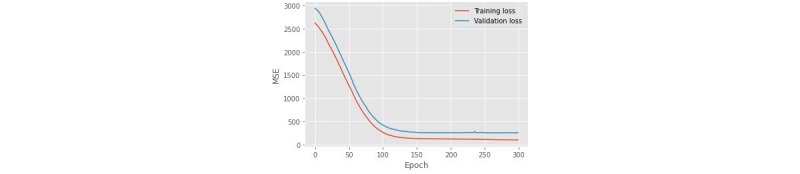
Training and validation loss of the long short-term memory model. MSE: mean squared error.

**Figure 3:**
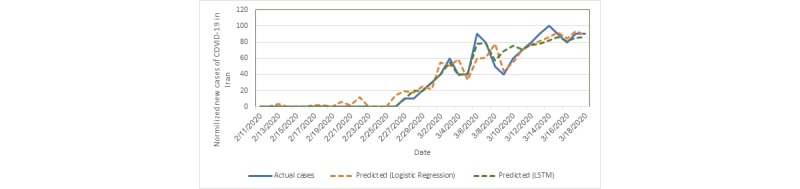
Actual and predicted new cases of COVID-19. LSTM: long short-term memory; COVID-19: coronavirus disease.

The features’ effect in the linear regression model is shown in Table 2. The RMSE for the linear regression model was 7.562 (SD 6.492). The LSTM RMSE was 27.187 (SD 20.705). The training and validation loss of the LSTM model is shown in Figure 2. The predictions made by these models are shown in Figure 3.

**Table 2:** Features’ effect on new daily cases in the linear regression model.

Feature	Coefficient (SE)	*t* value	*P* value
[Corona]_pd	–1.58 (0.77)	–2.05	.05
COVID-19_pd^a^	0.27 (0.12)	2.28	.03
Coronavirus_pd	1.55 (0.69)	2.26	.03
[Antiseptic selling] _pd	–0.09 (0.11)	–0.78	.44
[Antiseptic buying] _pd	0.32 (0.14)	2.33	.03
[Hand washing] _pd	0.44 (0.15)	3.01	.006
Hand sanitizer_pd	–2.01 (0.50)	–4.00	<.001
Antiseptic	1.52 (0.54)	2.80	.009
New cases_pd	1.03 (0.17)	6.05	<.001

^a^COVID-19: coronavirus disease

## Discussion

In this study, we proposed the use of prediction models for COVID-19 incidence in Iran using Google Trends data. Although the predictions are not very precise, they can be helpful for a base idea to build accurate models from more aggregation of data.

The features’ effect of the linear regression model shows that besides new cases in the previous day, hand sanitizer, handwashing, and antiseptic topics were the focus of the population. It could be inferred that people’s worries were increasing, and they were seeking prevention solutions. The lags in the prediction might have originated from other countries’ incidence. In other words, the population could be more sensitive and engaged in their health care after hearing news about the epidemic in other countries. This model could be used for other types of interventions such as assessing individuals’ awareness and engagement. Health authorities might use these data for measuring the information’s broadcast effect on the population and obtaining feedback from the search statistics.

The LSTM model showed a fluctuated performance in the folds while training loss was low. This indicates overfitting in the LSTM model because of the limited amount of training data. However, a low training error shows that the LSTM model can extract the pattern in the data. Therefore, we believe that by increasing the amount of training data, the LSTM model can outperform other models for this task. In addition, owing to the presence of just a few samples in each test fold (4 instances) and the subsequent high variation in RMSE, evaluation of the LSTM model was repeated with 3-fold cross-validation and the obtained RMSE was 13.45 (SD 7.90).

Past work on influenza and Zika virus predictions, for example, in the study by Santillana et al [13] in 2015, proposed a machine learning method for predicting influenza in the United States. In their study, the authors used data from Google searches along with Twitter data, hospital visit records, and a surveillance system. They provided multiple estimates to have an unbiased and more accurate prediction. They also showed that social media contains important information for effectively predicting disease incidence.

In 2017, McGough et al [14] also proposed a Zika virus prediction system by using Zika-related Google searches, Twitter microblogs, and a digital surveillance system. They also showed that the internet-based sources were useful to predict weekly Zika cases. In another related study in 2016, Majumder et al [15] used HealthMap surveillance data and Google Trends to predict cases of Zika virus in Colombia [15] and showed that digital surveillance data could be useful for the prediction of Zika cases. Further, in 2017, Teng et al [16] proposed a prediction model for Zika virus using search data from Google Trends and built the model using an autoregressive integrated moving average. They found a strong correlation between Zika-related searches and Zika cases. For the incidence prediction of influenza, socioenvironmental factors were considered when developing an epidemiological model named Susceptible Exposed to Infectious Recovered (SEIR) [17]. The model supports decision makers to factor the mass media and climate factors into the classical epidemic models. Another study emphasized the importance of environmental factors for the development of an influenza prediction model [18]. The findings of these studies along with our study show that internet resources could be helpful in pandemic forecasting.

The easy-to-obtain Google search data is a more dynamic and available source in comparison with traditional data sources. It could be a representation of the population’s thoughts, concerns, conditions, and needs in multiple periods. The major strength of this study is use of these data to predict the epidemiology of COVID-19 for the first time in the country.

In contrast, one major limitation of this study is the limited access to the Google Search data. Since Google Trends just provides data based on “interest” measure, more accurate and informative models could be built if the absolute search frequency is available for the researchers. It is worth mentioning that we used some of the keywords related to COVID-19 for extracting Google search frequencies; the selected keywords may have been incomplete and further research could aim to identify the most relevant set of keywords. In addition, future research should combine other data sources such as social media information, people’s contacts with the special call center for COVID-19, mass media, environmental and climate factors, and screening registries. Furthermore, in the broader context, such predictions could be made for other countries and even globally.

In conclusion, the data mining models could help policymakers and health managers to plan health care resources and control the prevention of an epidemic outbreak. The availability of high-quality and timely data in the early stages of the outbreak collaboration of the researchers to analyze the data could have positive effects on health care resource planning.

## Abbreviations

COVID-19: coronavirus disease
LSTM: long short-term memory
RMSE: root mean square error
SEIR: Susceptible Exposed to Infectious Recovered
WHO: World Health Organization
